# Small Fibre Neuropathy in Parkinson’s Disease: Comparison of Skin Biopsies from the More Affected and Less Affected Sides

**DOI:** 10.3233/JPD-191697

**Published:** 2019-10-11

**Authors:** Maria Jeziorska, Andrew Atkinson, Lewis Kass-Iliyya, Christopher Kobylecki, David Gosal, Andrew Marshall, Rayaz A. Malik, Monty Silverdale

**Affiliations:** a Division of Cardiovascular Sciences, University of Manchester, Manchester, UK; b Department of Neurology, Manchester Centre for Clinical Neurosciences, Salford Royal NHS Foundation Trust, Salford, UK; c Division of Neuroscience and Experimental Psychology, Manchester Academic Health Science Centre, University of Manchester, Manchester, UK; d Weill Cornell Medicine-Qatar, Doha, Qatar

**Keywords:** Parkinson’s disease, peripheral neuropathy, intraepidermal nerve fibre

## Abstract

We assessed small nerve fibre degeneration and regeneration in more and less affected sides in Parkinson’s disease (PD). Bilateral skin biopsies from 23 PD patients were immunostained for PGP9.5 for Intraepidermal Nerve Fibre Density (IENFD) and GAP-43 for mean axonal length (MAL), total epidermal (TNFL) and subepidermal nerve fibre length (SKTNFL). IENFD (*p* < 0.001) and SKTNFL (*p* < 0.001) were lower, whilst MAL (*p* < 0.001) and TNFL (*p* < 0.05) were higher in more affected versus less affected side. These results suggest increased small nerve fibre degeneration accompanied by enhanced nerve regeneration on the side more affected by PD and GAP-43 usefulness in skin biopsy assessment.

Parkinson’s disease (PD) is usually considered a central neurodegenerative process. However peripheral neuropathy (PN) is recognised as a feature of PD [1]. A recent systematic review in over 1300 PD patients showed large fibre PN in 16.3% and small fibre neuropathy in 56.9% of those who had a skin biopsy [2], compared to a 5.5% prevalence of PN in the general population [3]. Doppler et al found that the morphology of phosphorylated alpha synuclein and pattern of nerve fibre loss in skin biopsies were similar to changes seen in previous studies of substantia nigra pathology, leading them to postulate a common mechanism for peripheral and central neurodegeneration [4].

Intraepidermal nerve fibre density (IENFD) is the gold standard measure to quantify loss of skin innervation to diagnose a small fibre neuropathy (SFN) [5]. Several studies have demonstrated a significant reduction in IENFD in patients with PD [6–8]. These studies utilised immunostaining with the pan-axonal marker protein gene product 9.5 (PGP9.5). In our studies in patients with PD, also using PGP9.5, we have shown a reduction in IENFD and corneal small nerve fibre density and related it to autonomic dysfunction and the perception of affective touch [9, 10].

Recently, we have demonstrated the added value of applying more refined quantification of nerve fibre morphology which includes quantifying mean dendrite length (MDL) [11] and total nerve fibre length (TNFL) after immunostaining for growth associated protein-43 (GAP-43) [12], a marker of regenerating nerves in both experimental and human studies [13–15]. The MDL acronym was adopted from the literature, we now use a correct term MAL (mean axonal length) [16]. We have reported results of this technique in the PD population demonstrating both increased neurodegeneration and enhanced regeneration in PD versus controls [16].

It has been proposed that studying the peripheral neurodegenerative (and regenerative) process may help further understanding of the central neurodegenerative mechanisms in PD [4]. The central neurodegeneration in PD is asymmetrical [17, 18] and there is therefore interest in establishing the extent to which the peripheral neurodegeneration is also asymmetrical. Previous skin biopsy studies have established increased neurodegeneration on the more affected side, however these studies have only used PGP9.5 immunostaining therefore were not fully able to establish the asymmetry of enhanced regeneration [19, 20]. In the present study, we have undertaken morphological analysis of intraepidermal and sub-epidermal innervation in skin biopsies using immunostaining with PGP9.5 and GAP-43 in both more affected and less affected sides in patients with PD.

The study was approved by NRES committee/North West (Ref. No 12/NW/0086).

Thirty-three patients (22 males, 11 females) fulfilling the UK Brain Bank criteria for the diagnosis of Parkinson’s disease were recruited from neurology clinics. Ten patients (7 males, 3 female) were excluded after screening for other causes of peripheral neuropathy (cancer, chemotherapy, diabetes, impaired glucose tolerance, alcoholism, paraproteins, vitamin B6 and B12 deficiencies and autoimmune conditions). Unified Parkinson’s disease Rating Scale-III (UPDRS-III) was used to determine the more affected and the less affected side. Specifically, parts 3–8 (rigidity and bradykinesia scores) and parts 15–17 (tremor scores) were compared.

All 23 patients underwent 3 mm standard skin punch biopsies from the dorsum of each foot (more affected side [M] and less affected side [L]), 3 cm above the third metatarsal. Biopsies were processed as described previously [16]. The total length of nerve fibres in the epidermis (TNFL) and in the sub-epidermal skin layer (SKTNFL) normalised per millimetre length and mm^2^ area to provide standardised data (SKTNFL/Area; TNFL/Area; TNFL/Length and TNFL/BM [basement membrane]) were obtained. The mean length of nerve fibres crossing the BM into the epidermis (MDL) was measured on GAP-43 immunostained sections. These nerve fibre measurements were obtained for the more affected and less affected sides, and the percentage difference between the sides was calculated for each measurement.

GraphPad Prism v. 7 (GraphPad Software, Inc., USA) was used to perform all statistical analysis. The Shapiro-Wilk Test was used to assess the distribution of measurements. A two-tailed Wilcoxon matched-pairs signed rank test was used to compare means between the more affected and less affected sides for each intraepidermal and subepidermal nerve fibre nerve measurement. Data shown as mean (SD). *P* < 0.05 was taken to be significant. Additionally, Cohen d was calculated to measure effect size.

Table 1 indicates details of the study population. IENFD (no./mm) was 30% lower on the more affected (2.48±1.5, mean±SD) compared to the less affected (3.56±1.8) side, *p* < 0.001, d = –0.66. Nerve fibre branching was particularly evident on the more affected side, despite a lower IENFD. The length of nerve ramifications on the branches resulted in an increase in TNFL on the more affected side (Fig. 1A, B).

**Fig.1:**
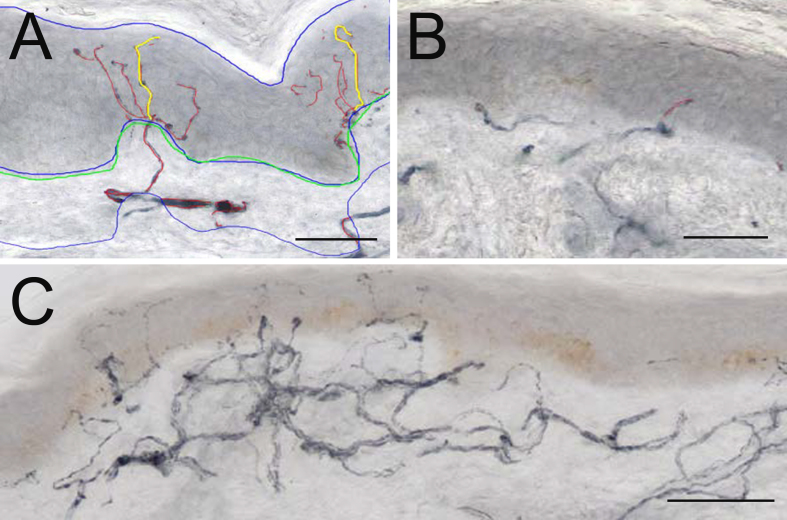
Representative examples of 50 μm sections from skin biopsies from more affected (M) and less affected (L) side from a PD patient, immunostained for GAP-43. Microphotograph from the more affected side (A) the blue tracings show area of epidermis and dermis; green line traces BM; red nerve fibres in epidermis and 50 μm subepidermal skin; yellow tracing shows nerve fibres measured for MAD. Note presence of branching nerves. Note closely positioned branching nerves. Panel B from less affected side shows area with short intraepidermal nerve fibres. Panel C shows a focal accumulation of nerves in subepidermal area which give rise to numerous NFs crossing the BM into epidermis. (Scale bars in A and B = 50 μm and in C = 100 μm).

**Table 1:** Demographics and clinical characteristics of PD patients

	PD patients
Gender	13 males, 10 females
Age	61.9±7.8
Disease duration (years)	6.4 (4.8)
UPDRS-III	25.96±12.7
Hoehn and Yahr stage	I = 10, II = 9, III = 4
Total cumulative levodopa dose (g)	*685.2 (118.8)

Data shown as mean±SD or *median (IQR) for normally and non-normally distributed data respectively.

MAL was 51% higher on the more affected (41.76±10.3) compared to the less affected (27.69±8.9) side, *p* < 0.001, d = 1.46. Mean TNFL/Length was 27% higher on the more affected (651.7±442.9) compared to the less affected (513.2±372.7) side, *p* < 0.05, d = 0.34 side. TNFL/Area was 27% higher on the more affected (8790±6553) compared to the less affected (6933±5138), *p* < 0.05, d = 0.32.

SKTNFL/Area was 30% lower on the more affected (16975±12023) compared to the less affected (23514±13486) side, *p* < 0.001, d = –0.51. A striking feature was the presence of isolated areas of large numbers of nerve fibre profiles in the papillary dermis (Fig. 1C) with an overall increase in SKTNFL on the less affected side.

TNFL/BM did not differ significantly between the more affected (462.1±289.6) and less affected (417.1±292.9) side, *p* = 0.3447, which confirmed visual observations of more flattened BM as part of more pronounced atrophic changes in the skin on the more affected side.

In this study we have quantified morphological measures of nerve fibre degeneration and regeneration in the epidermis and sub-epidermis to enable us to identify differences between the more affected and less affected side. We have incorporated the technique of GAP-43 immunostaining enabling us to quantify regeneration as well as degeneration. Huebner et al. provide strong evidence for GAP-43 being key to neurite outgrowth [15]. Overexpression of GAP-43 has been shown to promote axonal sprouting and terminal arborisation in rodents [21], whilst injection of GAP43 siRNA into DRG interrupts axonal regeneration [22].

Lauria et al. [23] investigated bilateral symmetrical skin punch-biopsies from lower extremities in patients with SFN and healthy volunteers using PGP9.5 for detection of nerve fibres and demonstrated high side-to-side correlation (R^2^ = 0.9608) of IENFD in both groups. Their overall conclusion was that the diagnosis of SFN can be reliably reached by unilateral skin biopsy at the distal site of the leg. The subsequent investigations of human peripheral innervation in different neuropathic conditions followed this advice.

Extensive search for bilateral skin biopsies in healthy individuals using GAP43 immunolocalisation did not yield any results. We support our choice of using nerve regeneration marker GAP43 for comparing innervation in symmetric bilateral skin biopsies in PD on the basis of the recent study localising small nerve fibres using double immunofluorescence for both PGP9.5 and GAP43 in SFN and healthy controls [24]. Admittedly, following Lauria recommendations they used single biopsies, but they clearly demonstrated that the ratio of GAP43/PGP9.5 measurements in control group was 0.93±0.13 in control group (similar age to our PD patients), demonstrating that in healthy skin, expression of both markers is very similar. Thus our demonstration of marked asymmetry in skin biopsy findings appears specific for PD.

There have been previous skin biopsy studies in PD, demonstrating reduced IENFD on the more affected side [19, 20]. However these studies have used only immunostaining for PGP9.5, not GAP-43. Here we have utilised GAP-43 immunostaining enabling a more refined analysis of the peripheral regenerative process in PD. Our results demonstrate lowered IENFD and SKTNFL on the more affected compared to less affected side. These markers are indictive of nerve fibre degeneration. We also demonstrated increased MAL, TNFL/Length and TNFL/Area on the more affected compared to less affected side. These markers are indicative of nerve fibre regeneration [11–15]. Thus we demonstrate both increased neurodegeneration and increased regeneration on the more affected compared to less affected side.

In our previous study of patients with PD using corneal confocal microscopy and skin biopsy, we have demonstrated that enhanced degeneration and regeneration is a pathological hallmark of Parkinson’s disease [9]. Podgorny and colleagues demonstrated SFPN in early drug naïve PD [25]. Nolano et al in a longitudinal study suggested that a reduced capacity for nerve regeneration with disease progression correlates with worsening symptoms and deficits [20]. Here we demonstrate the asymmetry of this process, which mimics the asymmetry of the central process. A pathological study of 21 patients with PD showed significant asymmetry with greater neuronal loss in the substantia nigra, which was contralateral to the initially affected body side [17]. It is thus tempting to speculate that a similar reduced capacity for regeneration over time may underlie the central neurodegenerative process.

The pathophysiology of small fibre neuropathy in PD is not yet clear. In particular we do not know whether the peripheral neurodegenerative process precedes the central process. Skin biopsy studies in prodromal PD will help to clarify this point. Interestingly, the severity of large fibre neuropathy is also a marker of PD severity [26]. However large fibre neuropathy in PD is associated with levodopa cumulative dose and altered homocysteine levels [27], which do not associate with small fibre neuropathy [19], suggesting a different pathological process.

A potential limitation of our study is the relatively small numbers of patients studied. However, these numbers are comparable to previous studies comparing the more affected with the less affected side. Nevertheless, we have utilised immunostaining with both PGP 9.5 and GAP-43 with detailed morphometric quantification to allow detailed assessment of small nerve fibre degeneration and regeneration. This has enabled us to demonstrate asymmetry of the peripheral neurodegenerative and regenerative processes which may mimic the asymmetry in central pathological mechanisms.

## CONFLICT OF INTEREST

The authors have no conflict of interest to report.

## References

[1] Toth C , Breithaupt K , Ge S , Duan Y , Terris JM , Thiessen A , Wiebe S , Zochodne DW , Suchowersky O (2010) Levodopa, methylmalonic acid, and neuropathy in idiopathic Parkinson disease. Ann Neurol 68, 28–36. 20582991 10.1002/ana.22021

[2] Zis P , Grunewald RA , Chaudhuri RK , Hadjivassiliou M (2017) Peripheral neuropathy in idiopathic Parkinson’s disease: A systematic review. J Neurol Sci 378, 204–209. 28566165 10.1016/j.jns.2017.05.023

[3] Hanewinckel R , Drenthen J , van Oijen M , Hofman A , van Doorn PA , Ikram MA (2016) Prevalence of polyneuropathy in the general middle-aged and elderly population. Neurology 87, 1892–1898. 27683845 10.1212/WNL.0000000000003293

[4] Doppler K , Ebert S , Uceyler N , Trenkwalder C , Ebentheuer J , Volkmann J , Sommer C (2014) Cutaneous neuropathy in Parkinson’s disease: A window into brain pathology. Acta Neuropathol 128, 99–109. 24788821 10.1007/s00401-014-1284-0 PMC4059960

[5] Lauria G , Hsieh ST , Johansson O , Kennedy WR , Leger JM , Mellgren SI , Nolano M , Merkies IS , Polydefkis M , Smith AG , Sommer C , Valls-Sole J , European Federation of Neurological Societies; Peripheral Nerve Society (2010) European federation of neurological societies/peripheral nerve society guideline on the use of skin biopsy in the diagnosis of small fiber neuropathy. Report of a joint task force of the European Federation of Neurological Societies and the Peripheral Nerve Society. Eur J Neurol 17, 903–912, e944-909. 20642627 10.1111/j.1468-1331.2010.03023.x

[6] Devigili G , Rinaldo S , Lettieri C , Eleopra R (2016) Levodopa/carbidopa intestinal gel therapy for advanced Parkinson Disease: AN early toxic effect for small nerve fibers? Muscle Nerve 54, 970–972. 27514937 10.1002/mus.25377

[7] Lin CH , Chao CC , Wu SW , Hsieh PC , Feng FP , Lin YH , Chen YM , Wu RM , Hsieh ST (2016) Pathophysiology of small-fiber sensory system in Parkinson’s disease: Skin innervation and contact heat evoked potential. Medicine (Baltimore) 95, e3058. 26962835 10.1097/MD.0000000000003058 PMC4998916

[8] Nolano M , Provitera V , Estraneo A , Selim MM , Caporaso G , Stancanelli A , Saltalamacchia AM , Lanzillo B , Santoro L (2008) Sensory deficit in Parkinson’s disease: Evidence of a cutaneous denervation. Brain 131, 1903–1911. 18515869 10.1093/brain/awn102

[9] Kass-Iliyya L , Javed S , Gosal D , Kobylecki C , Marshall A , Petropoulos IN , Ponirakis G , Tavakoli M , Ferdousi M , Chaudhuri KR , Jeziorska M , Malik RA , Silverdale MA (2015) Small fiber neuropathy in Parkinson’s disease: A clinical, pathological and corneal confocal microscopy study. Parkinsonism Relat Disord 21, 1454–1460. 26578039 10.1016/j.parkreldis.2015.10.019 PMC4671992

[10] Kass-Iliyya L , Leung M , Marshall A , Trotter P , Kobylecki C , Walker S , Gosal D , Jeziorska M , Malik RA , McGlone F , Silverdale MA (2017) The perception of affective touch in Parkinson’s disease and its relation to small fibre neuropathy. Eur J Neurosci 45, 232–237. 27859794 10.1111/ejn.13481

[11] Azmi S , Ferdousi M , Petropoulos IN , Ponirakis G , Alam U , Fadavi H , Asghar O , Marshall A , Atkinson AJ , Jones W , Boulton AJ , Tavakoli M , Jeziorska M , Malik RA (2015) Corneal confocal microscopy identifies small-fiber neuropathy in subjects with impaired glucose tolerance who develop type 2 diabetes. Diabetes Care 38, 1502–1508. 25877814 10.2337/dc14-2733 PMC4512140

[12] Culver DA , Dahan A , Bajorunas D , Jeziorska M , van Velzen M , Aarts L , Tavee J , Tannemaat MR , Dunne AN , Kirk RI , Petropoulos IN , Cerami A , Malik RA , Brines M (2017) Cibinetide improves corneal nerve fiber abundance in patients with sarcoidosis-associated small nerve fiber loss and neuropathic pain. Invest Ophthalmol Vis Sci 58, BIO52–BIO60. 28475703 10.1167/iovs.16-21291

[13] Denny JB (2006) Molecular mechanisms, biological actions, and neuropharmacology of the growth-associated protein GAP-43. Curr Neuropharmacol 4, 293–304. 18654638 10.2174/157015906778520782 PMC2475799

[14] Holahan MR (2017) A shift from a pivotal to supporting role for the growth-associated protein (GAP-43) in the coordination of axonal structural and functional plasticity. Front Cell Neurosci 11, 266. 28912688 10.3389/fncel.2017.00266 PMC5583208

[15] Huebner EA , Strittmatter SM (2009) Axon regeneration in the peripheral and central nervous systems. Results Probl Cell Differ 48, 339–351. 19582408 10.1007/400_2009_19 PMC2846285

[16] Jeziorska M , Atkinson AJ , Kass-Iliyya L , Javed S , Kobylecki C , Gosal D , Marshall A , Silverdale MA , Malik RA (2019) Increased intraepidermal nerve fiber degeneration and impaired regeneration relate to symptoms and deficits in Parkinson’s disease. Front Neurol 10, 111. 30837937 10.3389/fneur.2019.00111 PMC6383044

[17] Kempster PA , Gibb WR , Stern GM , Lees AJ (1989) Asymmetry of substantia nigra neuronal loss in Parkinson’s disease and its relevance to the mechanism of levodopa related motor fluctuations. J Neurol Neurosurg Psychiatry 52, 72–76. 2709038 10.1136/jnnp.52.1.72 PMC1032660

[18] Riederer P , Jellinger KA , Kolber P , Hipp G , Sian-Hulsmann J , Kruger R (2018) Lateralisation in Parkinson disease. Cell Tissue Res 373, 297–312. 29656343 10.1007/s00441-018-2832-z

[19] Nolano M , Provitera V , Manganelli F , Iodice R , Stancanelli A , Caporaso G , Saltalamacchia A , Califano F , Lanzillo B , Picillo M , Barone P , Santoro L (2017) Loss of cutaneous large and small fibers in naive and l-dopa-treated PD patients. Neurology 89, 776–784. 28747449 10.1212/WNL.0000000000004274

[20] Nolano M , Provitera V , Stancanelli A , Saltalamacchia AM , Caporaso G , Lullo F , Borreca I , Piscosquito G , Mozzillo S , Esposito M , Manganelli F , Lanzillo B , Santoro L (2018) Small fiber pathology parallels disease progression in Parkinson disease: A longitudinal study. Acta Neuropathol 136, 501–503. 29916036 10.1007/s00401-018-1876-1

[21] Aigner L , Arber S , Kapfhammer JP , Laux T , Schneider C , Botteri F , Brenner HR , Caroni P (1995) Overexpression of the neural growth-associated protein GAP-43 induces nerve sprouting in the adult nervous system of transgenic mice. Cell 83, 269–278. 7585944 10.1016/0092-8674(95)90168-x

[22] Xie W , Strong JA , Zhang JM (2017) Active nerve regeneration with failed target reinnervation drives persistent neuropathic pain. eNeuro 4, ENEURO.0008-17.2017. 10.1523/ENEURO.0008-17.2017 PMC5290455 28197545

[23] Lauria G , Dacci P , Lombardi R , Cazzato D , Porretta-Serapiglia C , Taiana M , Sassone J , Dalla Bella E , Rinaldo S , Lettieri C , Eleopra R , Devigili G (2015) Side and time variability of intraepidermal nerve fiber density. Neurology 84, 2368–2371. 25972491 10.1212/WNL.0000000000001666

[24] Bonhof GJ , Strom A , Puttgen S , Ringel B , Bruggemann J , Bodis K , Mussig K , Szendroedi J , Roden M , Ziegler D (2017) Patterns of cutaneous nerve fibre loss and regeneration in type 2 diabetes with painful and painless polyneuropathy. Diabetologia 60, 2495–2503. 28914336 10.1007/s00125-017-4438-5

[25] Podgorny PJ , Suchowersky O , Romanchuk KG , Feasby TE (2016) Evidence for small fiber neuropathy in early Parkinson’s disease. Parkinsonism Relat Disord 28, 94–99. 27160569 10.1016/j.parkreldis.2016.04.033

[26] Merola A , Rosso M , Romagnolo A , Comi C , Fasano A , Zibetti M , Lopez-Castellanos JR , Cocito D , Lopiano L , Espay AJ (2017) Peripheral neuropathy as marker of severe Parkinson’s disease phenotype. Mov Disord 32, 1256–1258. 28582598 10.1002/mds.27025

[27] Cossu G , Ceravolo R , Zibetti M , Arca R , Ricchi V , Paribello A , Murgia D , Merola A , Romagnolo A , Nicoletti V , Palermo G , Mereu A , Lopiano L , Melis M , Abbruzzese G , Bonuccelli U (2016) Levodopa and neuropathy risk in patients with Parkinson disease: Effect of COMT inhibition. Parkinsonism Relat Disord 27, 81–84. 27129930 10.1016/j.parkreldis.2016.04.016

